# miRNA-based regulation in growth plate cartilage: mechanisms, targets, and therapeutic potential

**DOI:** 10.3389/fendo.2025.1530374

**Published:** 2025-03-28

**Authors:** Prachi Thakore, Anne M. Delany

**Affiliations:** Center for Molecular Oncology, UCONN Health, Farmington, CT, United States

**Keywords:** microRNA, matrix vesicles, SOX9, regenerative medicine, miR-140, growth plate, chondrocyte, skeletal dysplasia

## Abstract

MicroRNAs (miRNAs) are critical regulators of the skeleton. In the growth plate, these small non-coding RNAs modulate gene networks that drive key stages of chondrogenesis, including proliferation, differentiation, extracellular matrix synthesis and hypertrophy. These processes are orchestrated through the interaction of pivotal pathways including parathyroid hormone-related protein (PTHrP), Indian hedgehog (IHH), and bone morphogenetic protein (BMP) signaling. This review highlights the miRNA-mRNA target networks essential for chondrocyte differentiation. Many miRNAs are differentially expressed in resting, proliferating and hypertrophic cartilage zones. Moreover, differential enrichment of specific miRNAs in matrix vesicles is also observed, providing means for chondrocytes to influence the function and differentiation of their neighbors by via matrix vesicle protein and RNA cargo. Notably, miR-1 and miR-140 emerge as critical modulators of chondrocyte proliferation and hypertrophy by regulating multiple signaling pathways, many of them downstream from their mutual target *Hdac4*. Demonstration that a human gain-of-function mutation in miR-140 causes skeletal dysplasia underscores the clinical relevance of understanding miRNA-mediated regulation. Further, miRNAs such as miR-26b have emerged as markers for skeletal disorders such as idiopathic short stature, showcasing the translational relevance of miRNAs in skeletal health. This review also highlights some miRNA-based therapeutic strategies, including innovative delivery systems that could target chondrocytes via cartilage affinity peptides, and potential applications related to treatment of physeal bony bridge formation in growing children. By synthesizing current research, this review offers a nuanced understanding of miRNA functions in growth plate biology and their broader implications for skeletal health. It underscores the translational potential of miRNA-based therapies in addressing skeletal disorders and aims to inspire further investigations in this rapidly evolving field.

## 1 Introduction

Longitudinal bone growth is regulated by a network of paracrine signals that sustain the unique structure and cellular dynamics of the growth plate. During this process, gene expression is tightly regulated by transcription factors and epigenetic mechanisms, including DNA methylation and chromatin remodeling, ensuring that key pathways are activated or repressed at the appropriate time. Beyond these controls of the transcriptional axis, non-coding RNAs, particularly microRNAs (miRNAs), play a pivotal role in the growth plate. miRNAs function as molecular rheostats that modulate mRNA stability and translation, adding regulatory robustness and the ability to fine-tune gene expression (1–3). miRNAs regulate cellular processes including differentiation, proliferation, apoptosis, and stem cell maintenance, impacting the expression of more than one-third of protein-coding genes. Thus, disruptions in miRNA activity are linked to numerous diseases, including skeletal dysplasias (4, 5).

**Figure 1:**
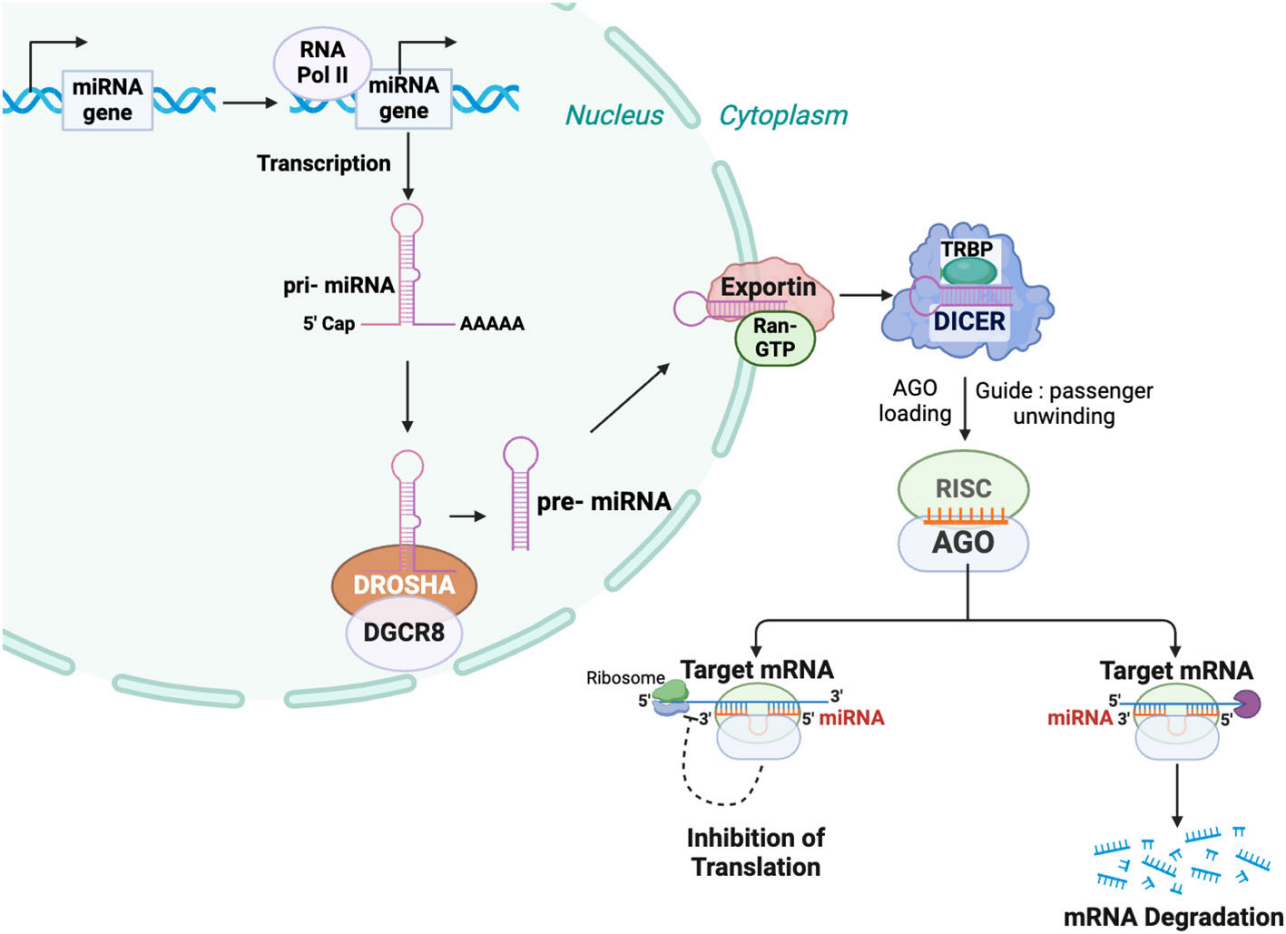
MicroRNA biogenesis and mechanism of function. In the nucleus, microRNA genes are transcribed primarily by RNA polymerase II (Pol II) into primary microRNAs (pri-miRNAs). These pri-miRNAs are processed by the Microprocessor complex, comprising Drosha ribonuclease III *(*DROSHA*)* and DiGeorge syndrome critical region 8 *(*DGCR8*)*, to produce precursor microRNAs (pre-miRNAs) of approximately 60–70 nucleotides. The pre-miRNAs are exported from the nucleus to the cytoplasm by an Exportin 5 (XPO5) containing complex. In the cytoplasm, the pre-miRNA is further processed by DICER, to generate a mature miRNA duplex. An Argonaute (AGO) family member is a key component of the miRNA-induced silencing complex (RISC), which unwinds the miRNA duplex and selects the miRNA guide strand. The miRNA-RISC complex binds to target messenger RNAs (mRNAs) via sequence complementarity, leading to mRNA degradation and/or translational repression.

First identified by the laboratories of Nobel laureates Victor Ambros and Gary Ruvkun, mature miRNAs are 18-23 nucleotide non-coding RNAs (6–8). A mature miRNA targets the RNA-induced silencing complex or RISC to complementary regions on mRNA transcripts, leading to their translational repression and/or degradation (**Figure 1**) (9). The complementarity of the miRNA seed region (nucleotides 2-8) with the target mRNA is crucial for initiating miRNA-mRNA interactions (10). The most efficient miRNA binding sites tend to be located within the 3’ untranslated region (UTR) of mRNAs, although there are reports of effective miRNA interactions within the 5’ UTR and coding region (11). There are thousands of miRNAs and each miRNA can potentially target hundreds of distinct mRNAs, making the understanding of their function a difficult but intriguing problem (5, 12).

The goal of this review is to provide a foundation for understanding the function of miRNAs in endochondral ossification and post-natal growth plate dynamics; we limit our presentation to individual miRNAs for which molecular mechanisms have been identified. While substantial research has been devoted to understanding the role of miRNAs in skeletal disorders such as osteoarthritis, we are not considering osteoarthritis here due to its broad scope and because it is a degenerative process rather than a skeletal dysplasia.

**Figure 2:**
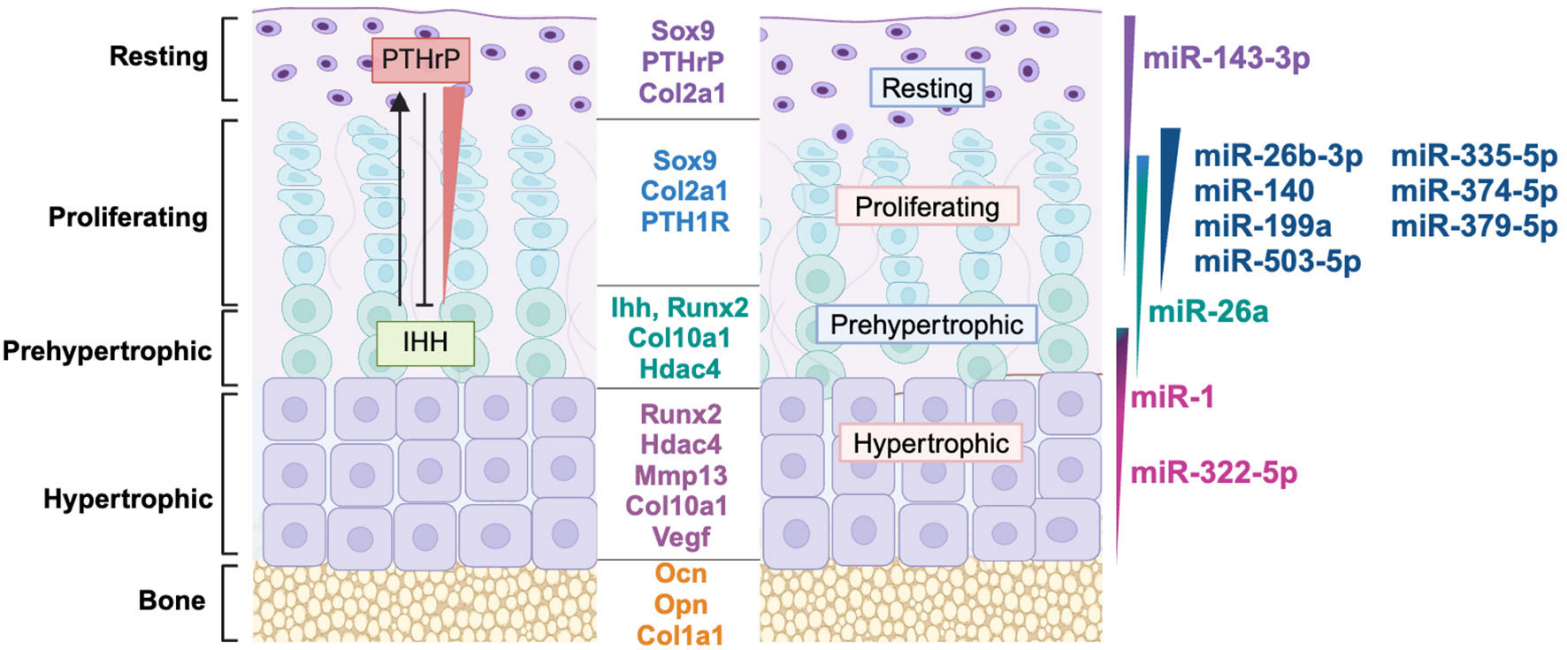
MiRNAs are differentially expressed in growth plate zones. Model of growth plate chondrocyte zones with their marker genes, key soluble signaling molecules and gradients of microRNA expression represented by varying colors across each zone. miRNA profiling data from (13) was used to determine the growth plate zone of expression. References for the function of miRNAs in this figure: miR-143-3p (14, 15), miR-26b-3p (16), miR-140 (17–20), miR-199a (21, 22), miR-503-5p (13), miR-335-5p (13, 23) miR-374-5p (13), miR-379-5p (13), miR-26a (24), miR-1 (25, 26), miR-322-5p (27, 28).

This review is based on a comprehensive search of peer-reviewed literature from PubMed (https://pubmed.ncbi.nlm.nih.gov/), focusing on studies with robust *in vivo* evidence and recent advancements in the field. The discussion of miRNAs is organized based on their activity within the distinct zones of the growth plate: resting, proliferating, and hypertrophic. However, due to the highly interconnected regulatory networks active in the growth plate, there is considerable overlap in miRNA functions and mRNA targets across the growth plate zones. Thus, it's reasonable to consider gradients of gene expression in both miRNAs and targets within the growth plate (**Figure 2**). Within the sections addressing specific growth plate zones, we first highlight miRNAs that have been most extensively studied *in vivo*, which may demonstrate therapeutic potential. Following this, we discuss less well studied miRNAs, with a particular emphasis on those investigated using *in vivo* models. For context, we first provide a broad overview of growth plate biology (Section 2), followed by a discussion of miRNA biogenesis and the broad impact of miRNAs in the growth plate (Section 3). Addressed next are miRNA function the resting/proliferating zones (Section 4) and in the proliferating/hypertrophic zones, including a discussion of miRNAs identified in skeletal dysplasias (Section 5). The review concludes with miRNAs found in cartilage matrix vesicles (Section 6), and a brief overview of miRNA-based therapeutics (Section 7).

## 2 Overview of growth plate biology

The axial and appendicular skeleton are predominantly formed via endochondral ossification, a process in which miRNAs play increasingly recognized regulatory roles. The developmental process of endochondral ossification begins with mesenchymal condensation at embryonic limb buds, where cells differentiate into chondrocytes that create a cartilaginous scaffold which is subsequently replaced by bone (29). Growth plates, located within these cartilage templates, are specialized structures that drive longitudinal bone growth and ossification, relying on a highly coordinated interplay of signaling pathways, gene expression, and miRNA regulation. Chondrocytes progress through distinct stages of proliferation, differentiation, hypertrophy and apoptosis, or to a limited extent, trans-differentiation into osteoblasts (30). **Figure 2** illustrates the distinct growth plate zones - resting, proliferative, prehypertrophic, hypertrophic - and the hallmark genes and signaling pathways active in each zone. Importantly, gene expression gradients are not limited to protein-coding genes but extend to miRNAs, which fine-tune the dynamic progression of chondrocytes through differentiation.

The resting zone contains slow-cycling chondrocyte and skeletal stem cells which are a source of cells for growth plate expansion. When resting zone chondrocytes are activated to proliferate, daughter cells organize into clonal cell columns which are characterized by the expression of key transcription factors, including SOX9, SOX5, and SOX6, as well as matrix proteins such as type II collagen (COL2A1) and aggrecan (ACAN) (31). Since SOX9 is essential for chondrogenesis, miRNAs targeting this transcription factor are of considerable interest.

As chondrocytes mature into prehypertrophic cells, they express Indian hedgehog (IHH) as well as PTH1R, which is the receptor for parathyroid hormone (PTH) and PTH-related peptide (PTHrP). The IHH-PTHrP feedback loop is a key mechanism maintaining the balance between proliferating and hypertrophic chondrocytes. Specifically, PTHrP, produced by resting zone chondrocytes, maintains chondrocytes in a proliferative state, and restrains premature hypertrophy in cells expressing its receptor PTH1R (32, 33). This ensures a sufficient pool of proliferating chondrocytes for continued growth. As chondrocytes move away from the influence of PTHrP, they begin expressing IHH, which signals their transition into hypertrophic cells. IHH then diffuses back through the growth plate, stimulating further PTHrP production and regulating the balance between proliferating and differentiating cells (34, 35). This feedback loop ensures that chondrocytes differentiate at an appropriate rate, preventing premature hypertrophy or imbalances that could impair growth plate function. By controlling both upstream and downstream targets within the PTHrP/IHH loop, miRNAs add an additional layer of precision to the feedback mechanism, emphasizing their role in tuning growth plate dynamics.

The mature hypertrophic chondrocytes then express the transcription factor RUNX2 and type X collagen (COL10A1), which are hallmarks of terminal hypertrophic differentiation. This transition to hypertrophy coincides with the downregulation of SOX9, SOX5, SOX6, and COL2A1 gene expression. As hypertrophic chondrocytes further differentiate, they begin expressing additional secreted molecules like VEGFA, MMP13, and SPP1 (secreted phosphoprotein 1/osteopontin), promoting the invasion of the region by endothelial cells, osteoclasts, and osteoblast precursors, leading to bone formation (36, 37).

In addition to the IHH-PTHrP feedback loop, chondrocyte differentiation is also modulated by a complex interplay of other signaling pathways including WNT, bone morphogenetic protein (BMP), transforming growth factor β (TGFβ) and their downstream effectors (TCF/LEF, SMADs, MAPKs) (38), as well as fibroblast growth factors (FGFs), with FGFR3 being crucial for regulating chondrocyte proliferation and hypertrophy (39). miRNAs targeting these essential signaling pathways, particularly the BMP pathway, also coordinate growth plate function.

Moreover, many miRNAs exhibit preferential expression in specific growth plate zones (**Figure 2**), where they regulate key genes and signaling pathways involved in chondrocyte maturation. **Table 1** summarizes the miRNAs discussed in this review, their validated gene targets, and their primary zones of expression. Taken together, the multidimensional regulation of gene expression and signaling pathways within the growth plate highlights its complexity. miRNAs, through their overlapping and spatially restricted functions, represent a regulatory layer that contributes to the robustness and adaptability of growth plate function; these intricate mechanisms will be explored in greater detail in subsequent sections.

**Table 1:** Key microRNAs documented in the growth plate, along with their primary zones of expression, functional roles, and specific gene targets.

MicroRNA	Primary Zone of Expression	Function	Gene targets related to chondrocytes/growth plate	Ref
mmu-miR-1	Hypertrophic	Regulates chondrocyte proliferation and inhibits hypertrophy	*Hdac4, Ihh*	(25, 26)
rno-miR-22-3p (matrix vesicle mRNA)	Resting and proliferating	Regulates IHH- PTHrP production and extracellular matrix formation	unknown	(40–42)
mmu-miR-26a	Proliferative and prehypertrophic	Inhibits hypertrophy and ECM protein synthesis	*Col10a1, Cd200*	(24)
hsa-miR-26b-3p	Proliferative	Inhibits chondrocyte proliferation and hypertrophy	*AKAP2*	(16)
rno/hsa miR-122-5p (matrix vesicle miRNA)	Resting	Promotes proliferation of resting and prehypertrophic chondrocytes, regulates ECM degradation	*Sirt1*	(41, 42)
hsa-miR-140	Proliferative	Regulates chondrocyte proliferation, hypertrophy and cartilage matrix formation	*HDAC4, ADAMTS5, DNEP, RALA*	(17–20)
rno-miR-143-3p	Resting and proliferating	Negatively regulates proliferation and cartilage matrix production	*Bmpr2*	(15)
mmu-miR-199a-3p	Proliferative	Inhibits chondrocyte differentiation	*Smad1*	(21)
mmu-miR-199a-5p		Inhibits chondrocyte hypertrophy	*Ihh*	(22)
rno miR-223-3p (matrix vesicle miRNA)	Resting	Increases chondrocyte proliferation	unknown	(40, 41)
mmu/hsa miR-322-5p	Prehypertrophic and hypertrophic	Promotes chondrocyte proliferation and differentiation	*Smad7, Mek1 Adamts5, Col12a1, Cbx6*	(27, 28)
rno/mmu-miR-335-5p	Proliferative	Promotes chondrocyte differentiation	*Dkk1, Rock1 and Daam1*	(23, 43, 44)
rno-miR-374-5p	Proliferative	Promotes chondrocyte proliferation and inhibits hypertrophy	unknown	(13)
rno-miR- 379-5p	Proliferative	Promotes chondrocyte proliferation and inhibits hypertrophy	unknown	(13)
rno miR-451-5p (matrix vesicle miRNA)	Resting	Increases chondrocyte proliferation	unknown	(41)
rno-miR-503-5p	Proliferative	Promotes chondrocyte proliferation and inhibits hypertrophy	*Sgk1*	(13)

miRNAs are listed in numerical order.

## 3 miRNA biogenesis and its impact on development and the growth plate

miRNA biogenesis is a multistep process initiated by the transcription of miRNA genes primarily by RNA polymerase II (**Figure 1**) (45, 46). Similar to an mRNA transcript, this primary miRNA (pri-miRNA) is capped and polyadenylated and can be hundreds or thousands of nucleotides long (47). Within the nucleus, the pri-miRNA is processed by complex containing the RNase III-type endonuclease DROSHA and DiGeorge syndrome critical region gene 8 (DGCR8) to a 60-80 base precursor miRNA (pre-miRNA) that retains a stem-loop structure (48). After being exported to the cytoplasm, it is further processed by a DICER-TAR RNA binding protein (TRBP) complex to an approximately 21-nucleotide miRNA duplex and then loaded from DICER to Argonaute (AGO) within the multiprotein RISC, where the guide strand remains associated with AGO while the passenger strand is degraded (49).

One of the earliest studies of miRNA function in mouse limb development involved conditional inactivation of the miRNA processing enzyme *Dicer* in the mesoderm lineage using T-cre (Tbxt-cre or Brachyury-cre), which is expressed at E6.5-E7.4 (50). Inactivation of *Dicer* in mesoderm at this stage increased cell death, which resulted in shorter somites in the paraxial mesoderm at E9.5, while paraxial mesoderm segmentation, itself, was not affected. However, the hindlimb bud, derived from the lateral plate mesoderm, was shifted posteriorly and the initiation of the limb bud formation was delayed. Normally, just prior to hindlimb bud formation, expression of the transcription factors *Hand2* and *Tbx3* is down regulated in the limb bud field. However, in the *T-cre-Dicer^f/f^* mice expression of *Hand2* and *Tbx3* was sustained, providing a potential mechanism for the shift in limb bud positioning. Both *Hand2* and *Tbx3* were subsequently shown to be targeted by miR-363 (50). Studies utilizing Prrx-cre to inactivate *Dicer* in mesoderm two to three days later, at E9.5, also showed increased cell death, but no defects in limb patterning per se. Still, the limbs were smaller and some morphological malformations were apparent in the *Prx-cre-Dicer^f/f^* mice (51). These studies suggest that the miRNA processing enzyme DICER is required for embryonic limb bud positioning and limb morphogenesis.

The first study documenting the essential role of miRNAs in endochondral ossification used *Col2a1-cre* to delete *Dicer* in the chondrocyte lineage. This caused a 60-70% decrease in the expression of most growth plate miRNAs, including the abundant let-7 family and miR-140. As a result, the *Col2-cre-Dicer^f/f^* mice exhibited severe skeletal growth defects and death just prior to weaning (52). The conditional *Dicer^-/-^* mice exhibited significantly impaired chondrocyte proliferation and disrupted progression to hypertrophic differentiation stages. Another study demonstrated the critical roles of DROSHA and DGCR8, the key components of the nuclear microprocessor complex, in cartilage development (53). Knockout of *Drosha* or *Dgcr8* in *Col2a1-cre* expressing cells severely disrupted miRNA biogenesis, resulting in reduced chondrocyte proliferation, premature hypertrophic differentiation, profound skeletal growth defects and perinatal lethality. Thus, although qualitatively similar, the phenotypes of *Dicer* deletion were somewhat milder compared to those resulting from the loss of *Drosha* or *Dgcr8*. While miRNA biogenesis was impaired in both the *Dicer* and *Drosha* knockout mice, the activity of these enzymes is not exclusive to miRNAs (53). It is possible that loss of some of these additional functions could have contributed to the differences in phenotypic severity among these models. Nonetheless, these studies underscore the impact of miRNAs on chondrocyte function, even though they do not address the function of specific miRNAs in the growth plate.

## 4 MicroRNAs expressed primarily in the resting and proliferating zones of the growth plate

### 4.1 miR-140

**Figure 3:**
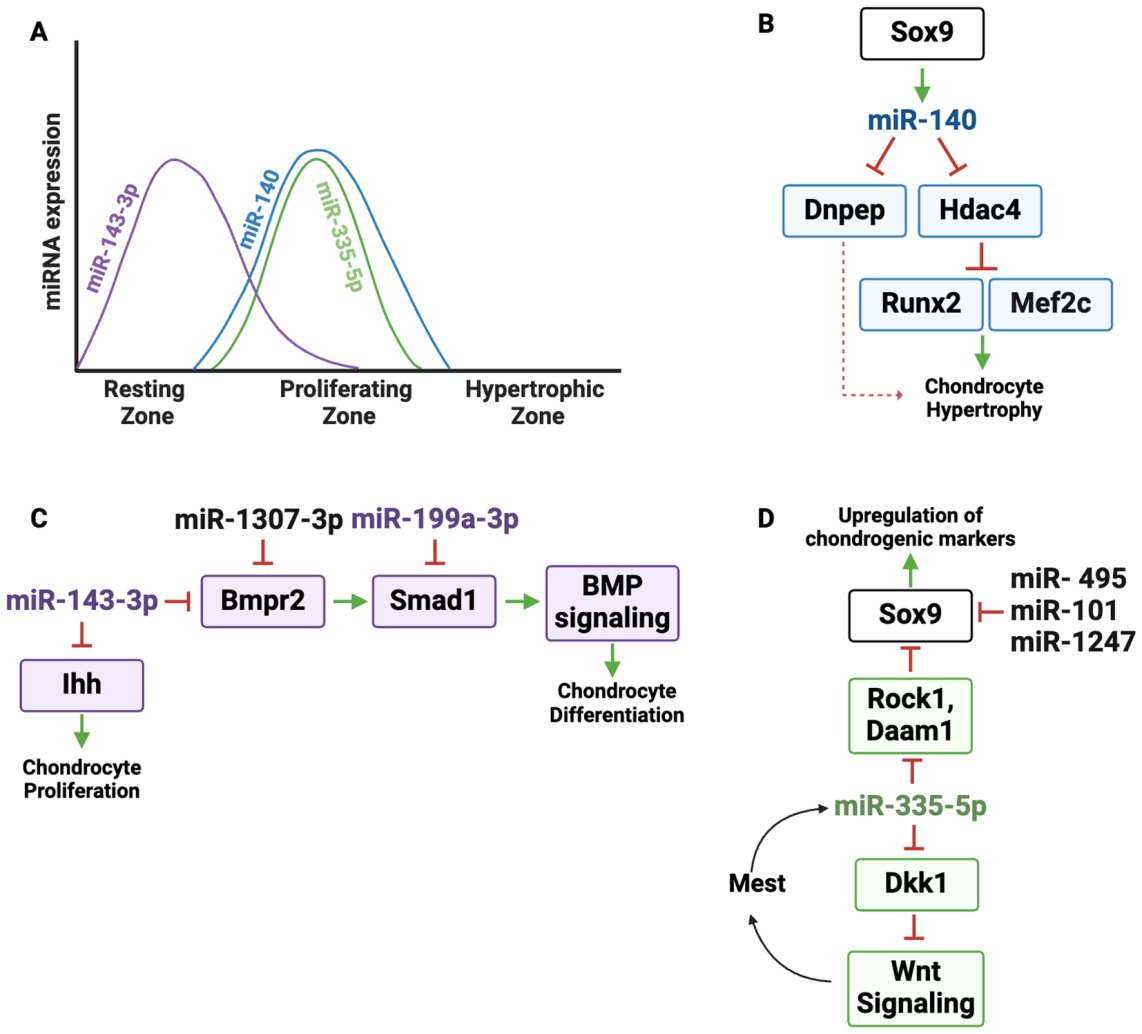
Regulatory landscape of miR-140 and other miRNAs expressed primarily in resting and proliferating zone chondrocytes. **(A)** Graphic depicting the dynamic expression of miR-140, miR-143-3p and miR-335-5p in the growth plate zones. **(B)** miR-140 is under the control of the master chondrogenic transcription factor SOX9. miR-140 directly targets *Dnpep* (aspartyl aminopeptidase*)* and *Hdac4*. HDAC4 suppresses the activity of the transcription factors MEF2C and RUNX2, which drive hypertrophic differentiation; *Dnpep* indirectly increases chondrocyte hypertrophy via dampening BMP signaling. **(C)** miR-143-3p targets *Bmpr2* and *Ihh.* By repressing *Bmpr2*, miR-143-3p dampens BMP signaling, reducing SOX9 activity and cartilage matrix production. IHH promotes chondrocyte proliferation, which could be limited by miR-143-3p expression. Since miR-1307-3p targets *BMPR2*, it can limit BMP signaling and chondrogenic differentiation. miR-199a targets *Smad1*, a SMAD that transduces signaling downstream of BMPR activation. **(D)** miR-335-5p supports chondrogenesis by targeting negative regulators of several pathways important for chondrogenesis. It directly represses RhoA-associated protein kinase (*Rock1*) and Dishevelled-associated activator of morphogenesis 1 (*Daam1*), both of which are suppressors of *Sox9*. Therefore miR-335-5p enhances SOx9 expression and upregulation of chondrogenic markers. miR-335-5p also targets Dickkopf-1 (*Dkk1*), an inhibitor of Wnt signaling. Therefore, miR-335-5p amplifies Wnt activity, further promoting chondrogenesis. Increased Wnt signaling promotes *Mest* transcription, which is the host gene for miR-335, boosting the expression of miR-335 in an amplification loop. miR-495, miR-101, and miR-1247 inhibit chondrocyte differentiation by targeting *Sox9*.

miR-140 is the first miRNA shown to be expressed in cartilage and is among the most abundantly expressed and extensively characterized miRNAs in chondrocytes (17). In the growth plate, miR-140 demonstrates a distinct expression pattern, predominantly in proliferating chondrocytes, as evidenced by RNA *in situ* hybridization. Its expression is notably minimal in hypertrophic chondrocytes, indicating a potential involvement in early stages of chondrogenic differentiation (**Figure 3A**) (17, 18, 54).

Global deletion of the Mir140 gene leads to a skeletal phenotype characterized by limb shortening, growth plate abnormalities and craniofacial deformities (18, 19, 55). miR-140-null mice display impaired differentiation of resting zone chondrocytes into columnar chondrocytes, while paradoxically accelerating the transition from proliferative to hypertrophic chondrocytes, leading to premature bone formation. However, despite this enhanced hypertrophic progression, the overall disruption in chondrocyte differentiation caused by miR-140 deficiency remains modest (19). Several studies outlined below delve into the broader mechanisms of miR-140 throughout the growth plate.

#### 4.1.1 miR-140 gene regulation and targets

Some miRNA genes are located in independent loci, while others are located within introns or exons of host genes (56). As an example of the latter category, the Mir140 gene is encoded within intron 16 of *Wwp2* and is co-expressed with the *Wwp2-C* isoform, suggesting a shared regulatory mechanism (57). miR-140 expression is controlled by the transcription factor SOX9, whose DNA binding and transactivation activity on the Mir140 promoter is enhanced by L-SOX5 and SOX6, two transcription factors also important for chondrocyte proliferation (58). The ability of SOX9 to promote miR-140 expression contributes to SOX9 function in the growth plate (**Figure 3B**) (18). For example, miR-140 targets histone deacetylase 4 *(Hdac4)*, a class IIa histone deacetylase (HDAC) that limits hypertrophy at least in part by negatively regulating the activity of transcription factors MEF2C and RUNX2, both of which are crucial for driving the transition of chondrocytes from proliferation to hypertrophy (17, 59, 60). MEF2C promotes hypertrophic differentiation by inducing expression of genes such as *Col10a1* (61), while RUNX2 serves as a master regulator of hypertrophic chondrocyte maturation and directly induces the expression of key markers, including *Ihh* and *Col10a1*, essential for terminal differentiation (62–64). By targeting *Hdac4*, miR-140 reduces its inhibitory effects on MEF2C and RUNX2, thereby indirectly promoting their function. Elevated levels of HDAC4 disrupt the expression of key matrix components such as *Col2a1, Col10a1*, and *Comp*, further underscoring the importance of miR-140 targeting in maintaining proper growth plate dynamics and extracellular matrix composition. Thus, the ability of miR-140 to target *Hdac4* plays a pivotal role in balancing chondrocyte differentiation by modulating downstream transcriptional regulators. In addition to HDAC regulation, miR-140 was shown to negatively regulate p38 MAPK signaling. Since p38 MAPK signaling is known to promote hypertrophic differentiation when overactivated, this illustrates another mechanism by which miR-140 may prevent premature chondrocyte maturation (55, 61, 65).

BMP signaling regulates the initiation of chondrogenesis *in vivo* and is critical for the expression of *Sox9, Sox5*, and *Sox6* genes (66). BMPs transduce signals by binding to heteromeric complexes of type 1 [BMPR1A, BMPR1B, or ActR1], and type 2 (BMPR2) serine/threonine kinase receptors, which activate downstream SMAD1/5/8 proteins to regulate gene expression (19, 66–68). miR-140 was shown to target aspartyl aminopeptidase *(Dnpep)*, a gene that modestly inhibits BMP signaling downstream of SMAD activation. In miR-140 deficient mice, elevated *Dnpep* levels reduce BMP activity, accelerating hypertrophic differentiation and causing skeletal defects (**Figure 3B**). Overall, targeting the miR-140/*Hdac4*/BMP axis could therefore present unique opportunities to treat skeletal dysplasias and prevent premature hypertrophy.

### 4.2 Other miRNAs targeting the BMP pathway, including miR-143-3p

While BMP signaling promotes the expression of key transcription factors such as SOX9, its activity must be carefully regulated to maintain the balance between proliferation and differentiation within the growth plate (67, 69). In the proliferative zone, BMP activity supports chondrocyte proliferation and extracellular matrix synthesis, while in the prehypertrophic and hypertrophic zones, BMP signaling drives differentiation and hypertrophy (66, 69). For instance, BMP2 enhances the expression of RUNX2 (70), and upregulates COL10A1, a hallmark of hypertrophic differentiation. Indeed, disruption of BMP signaling can result in severe skeletal abnormalities, including shortened long bones and disorganized growth plates, as seen in genetic models with impaired BMP receptor function (66).

miRNAs modulating components of the BMP pathway have the capacity to fine-tune cellular responses to BMP ligands in a zone-specific manner (**Figure 3C**). In one example, an RNA sequencing study comparing cells cultured from resting or prehypertrophic/upper hypertrophic zone of cartilage showed that miR-143-3p was highly expressed in the resting zone chondrocyte cultures (14). *In vitro* studies in rat mesenchymal cells showed that miR-143-3p targets *Bmpr2*, which is expressed throughout the growth plate (15, 71). miR-143-3p over expression *in vitro* decreased cartilage matrix production and decreased cell proliferation, an effect that could be related to its targeting of *Ihh* (72). Thus, miR-143-3p expression in resting zone cells likely decreases the sensitivity of these cells to BMP ligands, which could help maintain cells in the resting state. Conversely, decreased levels of miR-143-3p in prehypertrophic cells could relieve repression of its target *Ihh*, allowing expression of this key signaling component (**Figure 2**).

miR-199a-3p is another miRNA regulating the BMP pathway. miR-199a-3p is decreased during the BMP2-mediated chondrogenic differentiation of C3H10T1/2 cells and it targets *Smad1*, suggesting that miR-199a-3p is a negative regulator of differentiation (**Figure 3C**) (21). Likewise, miR-1307-3p inhibits the chondrogenic potential of human adipose-derived stromal/stem cells (hADSCs) by targeting BMPR2, emphasizing its inhibitory role in cartilage formation (**Figure 3C**) (73). Together, these miRNAs add a crucial layer of post-transcriptional control to the BMP axis, ensuring its integration into the complex spatial-temporal regulatory networks governing cartilage differentiation.

### 4.3 Other miRNAs targeting critical transcription factors and signaling pathways

Other *in vitro* studies describe miRNAs directly targeting *Sox9*, notably, miR-495, miR-101, and miR-1247 which act as inhibitors of chondrocyte differentiation (**Figure 3D**) (74–77). Inhibition of miRNAs such as these may be useful for promoting chondrogenic differentiation.

#### 4.3.1 miR-335-5p

The Mir335 gene is located within intron 2 of the *Mest* gene, and both miR-335-5p and *Mest* are increased during the chondrogenic differentiation of mesenchymal stromal cells (MSCs) *in vitro* (23, 78). miR-335-5p indirectly affects SOX9 expression by targeting the *Sox9* suppressors RhoA associated protein kinase (*Rock1*) and Dishevelled-associated activator of morphogenesis 1 (*Daam1*), providing mechanistic basis for increased *Sox9* expression and the upregulation of chondrogenic markers following miR-335-5p overexpression (**Figure 3D**).

In addition, miR-335-5p targets *Dkk1*, a secreted Wnt signaling inhibitor that binds to the LRP5/6 co-receptors to block WNT ligand binding (43). Although the role of WNT signaling in the growth plate is complex, it is thought to play a repressive role in chondrogenesis (79). By downregulating *Dkk1*, miR-335-5p amplifies WNT/β-catenin signaling, promoting *Mest* transcription and creating a positive feedback loop that boosts miR-335-5p expression and enhances its chondrogenic effects (**Figure 3D**) (23). The cartilage-enhancing effects of miR-335-5p, achieved by suppressing *Sox9* repressors and amplifying WNT signaling, underscore its therapeutic potential for cartilage repair and regeneration.

## 5 MicroRNAs with a major impact on the proliferating to pre-hypertrophic to hypertrophic zones of the growth plate

### 5.1 miRNA regulation by PTHrP

While synthesized primarily by resting zone chondrocytes, PTHrP acts on proliferating zone cells. A miRNA microarray demonstrated a handful of miRNAs regulated by PTHrP treatment during TGFβ-induced chondrogenic differentiation of hMSCs. Of these, miR-892b was modestly induced by PTHrP during the terminal phases of chondrogenic differentiation. Lentiviral-mediated over expression of miR-892b emulated the effects of PTHrP in that it enhanced expression of COL2A1 while inhibiting hypertrophy markers such as COL10A1 (80). miR-892b targets Kruppel-like factor 10 (KLF10/TEIG1), a transcription factor that directly binds to the IHH promoter region to drive transcription, providing potential mechanisms for limiting the premature synthesis of IHH in cells responsive to PTHrP. These investigators also demonstrated miR-892b targeting of *Wnt6*, a negative regulator of limb development, which may also play a role in regulating hypertrophy.

### 5.2 miR-374, miR-379 and miR-503

**Figure 4:**
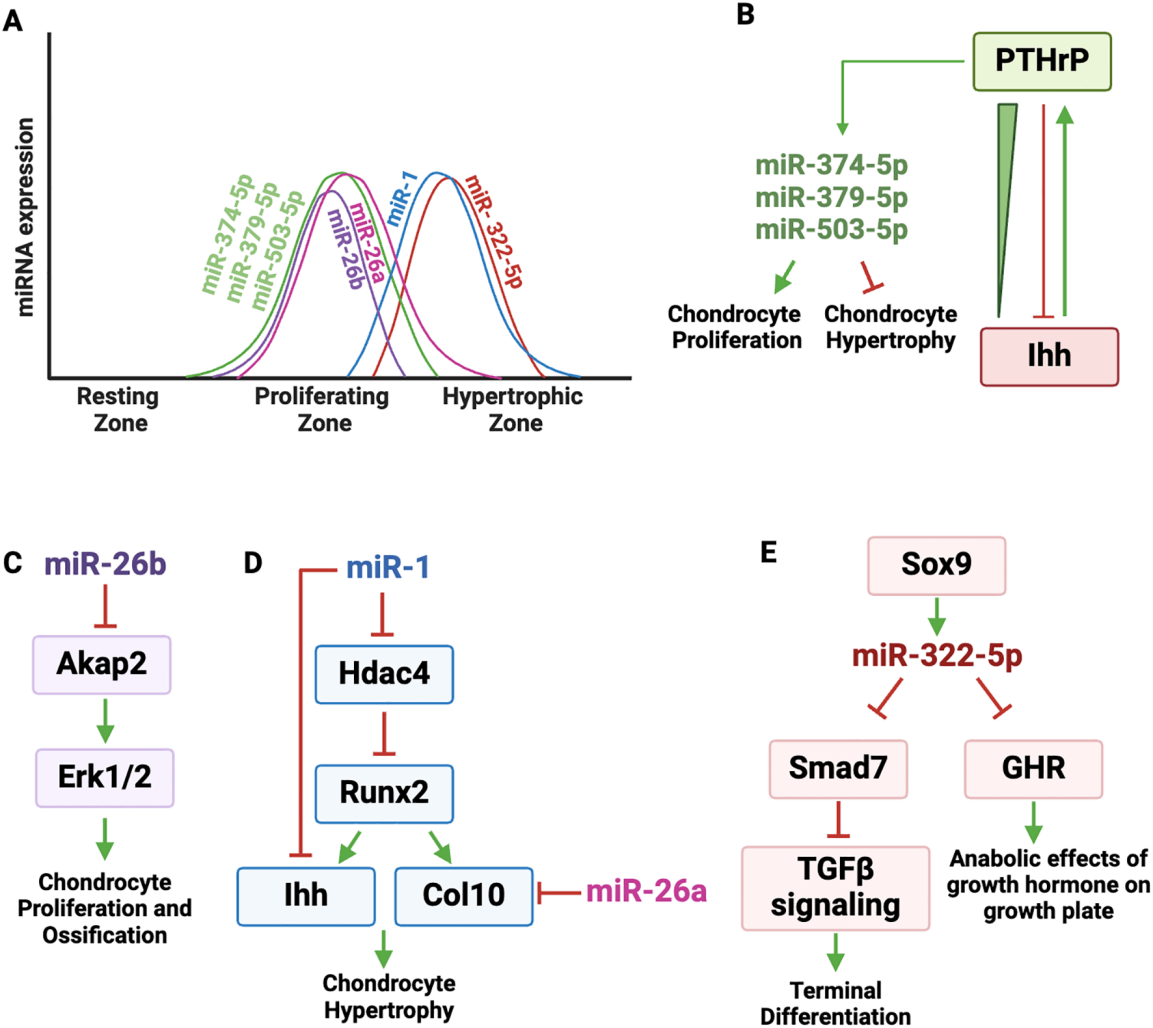
Regulatory landscape of miRNAs expressed in proliferating and hypertrophic zone chondrocytes. **(A)** Graphic depicting the dynamic expression of miRNAs active in proliferating and hypertrophic zones. **(B)** PTHrP and Ihh pathways form an important regulatory loop that controls chondrocyte proliferation and differentiation. PTHrP binds to its receptor PTH1R and induces the expression of miR-503-5p, miR-374-5p, miR-379-5p, which in turn promote chondrocyte proliferation and inhibit hypertrophic differentiation. **(C)** miR-26b targets A-Kinase Anchoring Protein 2 *(Akap2)*, a scaffold protein that anchors protein kinase A (PKA) and other signaling molecules. AKAP2 activity is important for the phosphorylation of ERK1/2, which is necessary for chondrocyte proliferation and ossification. **(D)** miR-1 directly targets *Hdac4 and Ihh*. HDAC4 suppresses RUNX2 transcriptional activity, which drives the expression of *Col10a1* and promotes hypertrophic differentiation. RUNX2 induces IHH expression. miR-1 targets *Ihh* and negatively affects chondrocyte hypertrophy. miR-26a targets *Col10a1*, negatively regulating extracellular matrix composition. **(E)** SOX9 increases the expression of miR-322-5p, which targets *Smad7*, a negative regulator of TGFβ signaling, which is needed for terminal differentiation. By targeting *Smad7*, miR-322 helps promote terminal differentiation of chondrocytes. miR-322-5p also targets *GHR* (growth hormone receptor), which is expressed in the resting and proliferating zones of the growth plate and mediates the anabolic effects of growth hormone on the growth plate.

A robust miRNA profiling study conducted on micro-dissected zones of the rat growth plate identified differential expression of 34 miRNAs between the proliferative and hypertrophic zones (13). Out the 29 highly expressed miRNAs in the proliferative zone, several miRNAs (miR-503-5p, miR-374-5p, miR-379-5p) were prioritized for further study, based on their potential to target a panel of established markers of hypertrophic differentiation (**Figures 4A, B**). The authors hypothesized that expression of these miRNAs could contribute to proliferation, delay hypertrophy and may be induced by PTH1R signaling. Indeed, inhibition of miR-503-5p, miR-374-5p or miR-379-5p in rat primary chondrocytes decreased proliferation and stimulated hypertrophic differentiation specifically affecting *Ihh*, *Bmp2* and *Bmp6* expression. Further, these miRNAs were induced by PTH1R signaling in rat growth plate chondrocytes *in vitro* (**Figure 4B**). The authors proposed a mechanistic model in which the PTHrP concentration gradient across the growth plate zones increases the expression of miR-503-5p, miR-374-5p, and miR-379-5p, which in turn promotes chondrocyte proliferation and inhibits hypertrophic differentiation. This work highlights the interaction between PTHrP signaling and miRNA-mediated gene regulation in growth plate.

### 5.3 miR-26 family

The miR-26 family is composed of miR-26a and miR-26b, which are highly conserved across species and have overlapping but distinct roles in regulating key biological pathways, particularly in bone and cartilage development (81).

#### 5.3.1 miR-26a

In qPCR analysis of microdissected growth plate cartilage, miR-26a expression was observed across the proliferative and prehypertrophic zones, but was consistently downregulated in the hypertrophic zone (**Figure 4A**). miR-26a was shown to target hypertrophic marker genes *ColXa1* and *Cd200* (**Figure 4D**) (24, 82). Although miR-26a does not directly target extracellular matrix adaptor proteins like matrilin-3 and *COMP* (83), their expression was significantly reduced in cells transfected with miR-26a mimics, suggesting both direct and indirect effects of miR-26a on the extracellular matrix composition (84).

#### 5.3.2 miR-26b-3p

One of the most interesting and recent findings emphasizes the involvement of miR-26b-3p in idiopathic short stature (ISS), a condition characterized by impaired bone growth with no clear etiology (16). In this study, exosomes isolated from the plasma of juvenile ISS patients were found to inhibit chondrocyte proliferation and differentiation *in vitro*. Subsequent miRNAseq analysis of plasma exosomes from these children revealed miR-26b-3p to be the most highly up regulated miRNA in ISS patient exosomes, so much so that plasma exosome miR-26b-3p levels could distinguish ISS patients from healthy individuals. In human chondrocytes, over expressing miR-26b-3p led to a marked reduction in growth and reduced expression of critical proteins linked to endochondral ossification, including RUNX2, COL10A1, osteocalcin, and osteopontin.

The molecular mechanism underlying miR-26b-3p action was shown to involve its targeting of A-Kinase Anchoring Protein 2 *(Akap2)*, a scaffold protein that anchors protein kinase A (PKA) and other signaling molecules, playing a key role in cellular processes such as proliferation and differentiation (85). AKAP2 activity is important for the phosphorylation of ERK1/2, and p-ERK1/2 activity is necessary for both chondrocyte proliferation and subsequent ossification (**Figure 4C**).

*In vivo*, overexpression of miR-26b-3p in rats via adenovirus or exosome-mediated delivery confirmed its inhibitory effect on growth plate chondrocytes, resulting in decreased *Akap2*, fewer proliferative cells in the growth plate and diminished expression of hypertrophy and ossification markers. These findings suggest that miR-26b-3p may play a role in the pathogenesis of ISS; therapeutic targeting of miR-26b-3p may offer the potential to restore normal chondrocyte function and bone growth in ISS patients. Moreover, since nearly a quarter of children initially diagnosed with ISS have an underlying genetic etiology or skeletal dysplasia affecting the growth plate, miRNA profiling could improve diagnostic precision, particularly where traditional growth hormone therapy shows limited effectiveness (86, 87).

### 5.4 miR-1

miR-1 is primarily expressed in the pre-hypertrophic and hypertrophic zones of the growth plate (**Figure 4A**). It has a complex genomic organization, as the Mir1 gene is clustered with Mir133 on two different mouse chromosomes and both Mir1 genes give rise to the same mature miRNA (25). For example, on chromosome 2, Mir1-2 and Mir133a-1 are separated by 9.3 kb and may not be regulated together. In contrast, on chromosome 18, the Mir1-1 and Mir133a-2 genes are separated by 2.5 kb, appear to be within the same pri-miRNA, and both miR-1 and miR-133 are enriched in matrix vesicles (88). miR-1 and miR-133a provide an example of how multiple miRNAs may be co-transcribed within a single pri-miRNA, enabling coordinated expression, although miR-133a targets in the growth plate have not been examined.

As previously discussed, HDAC4 is a negative regulator of chondrocyte hypertrophy in part because it interacts with and inhibits RUNX2 activity, and miR-1 directly targets an evolutionarily conserved region of the *Hdac4* 3’ UTR (**Figure 4D**) (25). By targeting *Hdac4*, miR-1 reduces the inhibitory effect of HDAC4 on RUNX2, resulting in increased expression of the RUNX2 target genes *Col10a1* and *Ihh in vitro* (60, 89). A subsequent *in vitro* study showed direct targeting of *Ihh* by miR-1 (90), while transgene-mediated overexpression of miR-1 in *Col2a1* expressing cells led to shorter limb and body length, delayed formation of secondary ossification centers and delayed terminal differentiation (26). The Col2a1-miR-1 transgenic mice displayed reduced *Ihh* expression in growth plate chondrocytes both *in vivo* and *in vitro*, with decreased expression of key hypertrophic markers such as *Runx2, Col10a1, and Mmp13*, further impacting chondrocyte hypertrophy. Taken together, these findings suggest that miR-1 is involved in an incoherent regulatory network, modulating both stimulatory and inhibitory factors of hypertrophy (**Figure 4D**).

### 5.5 miR-322-5p

In mice, the gene coding for miR-322-5p is located within a miRNA cluster on the X chromosome (Mirc24 cluster contains Mir351, Mir503, Mir322) (27). While miR-322 is not found in humans, its human ortholog is miR-424, which is also located on the X-chromosome within a miRNA cluster, along with MIR503. Since work on miR-322/miR-424 function in cartilage has been performed mainly in rodents, our discussion here focuses on miR-322.

miRNA-322-5p is well expressed in the prehypertrophic and hypertrophic zones in mice and during the terminal differentiation of a mouse chondrogenic cell line *in vitro* (**Figure 4A**). While the majority of miRNAs function as negative regulators of RNA stability and translation, the first studies on miR-322-5p in growth plate showed that this miRNA inhibits the RAF/MEK/ERK pathway in cartilage cells by stabilizing *Mek1* RNA. Deletion of the Mirc24 cluster in *Col2a1* expressing cells *(Col2a1-Cre-Mirc24tm1M)* led to shortened hypertrophic zone length, decreased protein levels of SOX9 and SOX6, and corresponding decreases in *Acan* and *Col10a1* levels (27, 91).

A recent *in vitro* study showed that Sox9 increases the expression of miR-322-5p, which targets *Smad7*, a negative regulator of TGFβ signaling (**Figure 4E**) (92). The effect of TGFβ signaling in the growth plate is dependent on chondrocyte differentiation stage. For example, deletion of *Tgfbr2* in Prx-cre expressing cells results in decreased proliferation, accelerated transition from prehypertrophic to hypertrophic, and delayed late hypertrophic differentiation (93, 94). Since miR-322-5p is expressed in the prehypertrophic and hypertrophic zone cells, it is possible that this miRNA functions to promote hypertrophic differentiation, as it targets a negative regulator of TGFβ signaling (95) (**Figure 4E**).

More translational studies in rats linked miR-322-5p levels in blood exosomes with skeletal size in pups with low birth weight caused by fetal malnutrition (96). Specifically, low birth weight pups that failed to catch up in size after birth had increased levels of miR-322-5p in their liver and in blood exosomes. miR-322-5p levels correlated inversely with body length and growth hormone receptor levels. Subsequently, miR-322-5p was shown to target the growth hormone receptor (*GHR*), which is expressed in the resting and proliferating zones of the growth plate and mediates the anabolic effects of growth hormone on the growth plate (**Figure 4E**) (97, 98). miR-322-5p is not abundantly expressed in resting and proliferating zone cells, and these studies support the concept that miRNAs from other tissues, such as liver, delivered to the skeleton by exosomes, could have a paracrine function on growth plate activity.

Additionally, research into pregestational diabetes mellitus revealed that maternal hyperglycemia can restrict fetal endochondral bone growth, causing a marked reduction in the length of the hypertrophic zone and decreased miR-322-5p expression in this region (28). Complementary *in vitro* experiments confirmed that high glucose exposure downregulated miR-322-5p in a chondrogenic cell line, impacting critical markers like *Sox9* and *Pthlh*, which maintain proliferative chondrocytes and delay hypertrophy. Simultaneously, the expression of hypertrophic markers *Runx2* and *Col10a1* was increased. While this work highlights the potential impact of glucose levels on miRNAs in the growth plate, there is still much to be learned about the miRNA-mRNA target interactions underlying this growth plate pathology. Further, while the study suggests a role for altered miR-322-5p expression in hyperglycemia, the mechanisms by which glucose or metabolic hormones may regulate miR-322 levels remain undetermined.

### 5.6 miRNA in skeletal dysplasia

#### 5.6.1 miR-140 gain-of-function mutation

A pivotal study reported the first known pathogenic neomorphic gain-of-function miRNA mutation, which was identified in the *MIR140* gene, miR-140-5p-G; this mutation was linked to a novel form of autosomal dominant human skeletal dysplasia (99). Affected individuals exhibit skeletal abnormalities including spondylar dysplasia, delayed bone ossification, brachydactyly, and short stature. Modeling of this mutation in mice showed efficient production of the mutant miR-140-5p-G, causing both derepression of wild-type targets and repression of new miR-140-5p-G-specific mRNA targets, thus demonstrating a dual gain- and loss-of-function mechanism. For example, the mutant miR-140-5p-G could target *Hif1a*, among other transcripts functional in cartilage. These alterations in gene expression resulted in delayed secondary ossification center formation, decreased *Col10a1* and delayed skeletal maturation. This discovery highlights the complex regulatory role of miRNA mutations in human disease.

#### 5.6.2 miR-17~92 cluster

The miR-17~92 cluster is conserved across species and encodes six miRNAs (miR-17, miR-18a, miR-19a, miR-20a, miR-19b1, and miR-92a1) (100). Deletion mutations in the MIR17HG gene, which encodes this miRNA cluster, are linked to skeletal defects in humans, including Feingold syndrome type 2, which is characterized by developmental delay, short stature, and multiple skeletal abnormalities, including brachysyndactyly (101). Conversely, one case study of a 9-year-old boy with developmental delay and skeletal abnormalities revealed a microduplication at 13q31.3, affecting the MIR-17HG gene (102). Overall, these data suggest that gene dosage imbalances in this cluster can lead to a spectrum of developmental outcomes, underscoring the complex role of the miR-17~92 cluster in skeletal development.

The miRNAs encoded in the Mir17~92 cluster are also contained within two additional paralogous gene clusters, Mir106a~363 and Mir106~25 (103). Because of this, mouse studies addressing the function of the miR-17~92 cluster in growth plate were performed in compound mutant mice in which Mir17~92 was deleted in Prx1 expressing cells *(Prx1-Cre: Mir17-92^f/f^)*, and Mir-106b-25 was deleted globally (miR-106b-25^-/-^) (104). The phenotype of these compound mutant mice recapitulated many of the skeletal abnormalities seen in Feingold syndrome type 2 patients. Mechanistically, miR-17 was shown to target *Tgfbr2*, and TGFβ signaling was increased in limb bud cultures from the compound mutant mice lacking the miR-17~92 and miR-106b-25 clusters. Decreasing TGFβ signaling in the mutant mice, either by genetic means or by treatment with a TGFβ receptor inhibitor in the early embryonic stages partially rescued skeletal abnormalities. This work illustrates the utility of comprehensively understanding specific miRNA-target interactions and how this understanding can be translated into important proof-of-concept studies on novel therapeutic strategies.

## 6 miRNAs enriched in matrix vesicles, affecting differentiation and proliferation of chondrocytes

Matrix vesicles are extracellular matrix-bound exosomes ranging from 50 to 150 nm in diameter. While exosomes and matrix vesicles have some common features, matrix vesicles are enriched in certain lipid and protein components important for their ability to bind matrix and for nucleation of mineralization (105–107). Matrix vesicles contain minerals, enzymes, factors, and miRNAs. Since growth plate lacks vascularization and chondrocytes are surrounded by extracellular matrix, matrix vesicles may be a means for cells to influence the function and differentiation of their neighbors by via their protein and RNA cargo (105).

Due primarily to the difficulties in isolating them from the extracellular matrix, matrix vesicles are challenging to study *in vivo*. Therefore, much of the work on matrix vesicles in the growth plate has been performed using cultured chondrocytes. Such studies have shown that the miRNA composition of matrix vesicles isolated from cultured resting zone chondrocytes differs from those isolated from cultures of growth zone chondrocytes (14, 40). Moreover, the abundance of miRNAs within chondrocyte matrix vesicles can differ notably from those in their parent chondrocyte cells, reflecting the fact that some miRNAs are preferentially packaged into exosomes (14, 40, 41, 108).

In miRNA-seq analysis of matrix vesicles from cultured rat chondrocytes derived from either the resting zone or growth zone, miR-142-3p was the most abundant miRNA species identified, accounting for about 30% of the reads. miR-21-5p and miR-22-3p were also highly abundant, and the abundance of these species reflected their general abundance in the parent chondrocytes (40). Transfection of cultured rat chondrocytes with miR-22-3p mimic resulted in decreased proliferation and proteoglycan content and upregulated PTHrP and IHH, supporting functions in both resting and growth zone cells (41).

MiRNAs enriched in resting zone matrix vesicles include miR-150-5p, miR-451-5p, miR-223-3p, miR-142-3p, and miR-122-5p (14, 40). Transfection of chondrocytes with miR-451-5p or miR-223-3p mimics impacted cell proliferation, but did not affect proteoglycan production or the expression of IHH or PTHrP. In contrast to miR-22-3p, chondrocytes transfected with miR-122-5p mimic displayed increased proliferation and proteoglycan content, and decreased *Ihh* and *PTHrP* production, suggesting an inhibition of differentiation (41, 42). In follow up studies, RNAseq analysis was performed on chondrocytes transfected with miR-22-3p or miR-122-5p mimics, and differential gene expression data were reported. These studies could provide some mechanistic insight into the function of miR-22-3p and miR-122-5p in the different zones of the growth plate (42).

## 7 Recent advances in miRNA-based therapeutics

### 7.1 Proof-of-concept studies

A recent proof-of-concept study explored the therapeutic potential of targeting miR-338-3p in the treatment of cleidocranial dysplasia (CCD), a genetic bone disorder caused by decreased expression or activity of RUNX2, and characterized by skeletal abnormalities, including short stature and osteopenia, as well as anomalies involving the axial skeleton, skull and teeth (109, 110). Earlier work demonstrated that *Runx2* is targeted by miR-338-3p, a miRNA found to be increased in the serum of post-menopausal osteoporosis patients and in mice post-ovariectomy (111). Using mice haploinsufficient for *Runx2* as a CCD model, knockout of the *Mir338~3065* cluster was able to rescue much of the bone growth defect caused by decreased *Runx2* levels, whereas rescue of the skull defects was less prominent (109). Overall, this work suggests that decreasing the expression of miRNAs that target RUNX2 may be a strategy for ameliorating some of the skeletal phenotypes caused by CCD.

### 7.2 Overlapping mechanisms between articular cartilage repair and the growth plate

Most studies addressing the potential of miRNA-based therapeutics for cartilage disorders have been performed in the context of arthritis, a multifaceted disease impacting articular cartilage and the underlying bone. This is understandable, since the prevalence of arthritis is very high, particularly osteoarthritis, and is associated with significant impacts on quality of life, posing an important economic burden. Moreover, since skeletal stem and progenitor cells are becoming more therapeutically relevant for degenerative musculoskeletal diseases, an understanding of how miRNAs may regulate the persistence of stem-like properties and commitment to specific lineages is of immense interest. In this regard, studies have documented miRNA expression profiles during the *in vitro* chondrogenic differentiation of mesenchymal stem cells and validated some potential targets of differentially regulated miRNAs. Among the miRNAs reviewed herein, these studies have highlighted functionality for miRNAs including miR-140-5p, miR-143-3p, and miR-199a (21, 112, 113). Another study established a panel of miRNAs that might be useful for determining whether MSCs from human donors may have low or high chondrogenic potential *in vitro*, which could have an impact on selecting donor cells for use in tissue regeneration applications (114). Fortunately, many of signaling mechanisms and regulatory pathways are highly conserved between articular cartilage and growth plate cartilage (71, 115), so insights into miRNA function in growth plate may be informative for articular cartilage, and some of the more translational work on miRNA-based therapeutics for osteoarthritis may be relevant to the management of growth plate injury.

Much of the work on miRNAs in osteoarthritis has focused on pathways involved in articular cartilage matrix degradation, chondrocyte apoptosis and inflammation. Since miRNAs can act as both promoters and suppressors within these processes, therapeutic potential may be realized through administration of either synthetic miRNA inhibitors (antagomirs; anti-miRs) or mimics (agomirs) (116). Because inflammation, as well as cartilage matrix degradation, is a hallmark of osteoarthritis, miRNAs targeting pro-inflammatory cytokines and signaling molecules are of particular interest. miR-140, for example, is downregulated in human osteoarthritis cartilage samples compared with healthy controls (117). Transfection of chondrocytes with miR-140 mimic antagonized the ability of IL1β to increase expression of the matrix degrading enzyme *Adamts5* and the IL1-mediated decrease in *Acan* (118). Subsequent studies demonstrated that joint destruction was attenuated in transgenic mice expressing miR-140 under the control of the *Col2a1* promoter, providing proof-of-concept for a potential miR-140 mimic-based therapeutic. Moreover, targeting of *Adamts5* by miR-140 provides a mechanism contributing the protective effect of miR-140 on matrix degradation (18). Extensively studied in both osteoarthritis and growth plate, the role of miR-140 in maintaining both articular cartilage and growth plate function highlights its therapeutic potential. For additional information on miRNA function in osteoarthritis, please consider this excellent review (119).

### 7.3 Physeal fractures and bony bridge formation

Beyond osteoarthritis, the potential of miRNA-based therapeutics for addressing cartilage injuries could be explored in an a scenario specifically relevant to the growth plate: the treatment of physeal bony bridge or bony bar formation post-injury in pediatric patients (120). Physeal bars occur most commonly following trauma or fracture. They extend through the growth plate between the epiphysis and metaphysis, resulting in an abnormal bony repair tissue that bridges the two regions. This bony bridge disrupts growth plate function, leading to growth arrest, angular deformities, and limb length discrepancies due to impaired longitudinal bone growth. Thus, it represents a critical issue in pediatric orthopedic care (121).

Physeal injury is fairly common, comprising 15-30% of pediatric fractures. Of these, an estimated that 1-10% of physeal injuries result in bony bridge formation (122). The probability of poor outcome increases with the proportion of the growth plate affected. For example, fractures most likely to demonstrate physeal bar formation are those with fracture through the physis into the epiphysis or those in which the fracture extends from the articular surface to the metaphysis (123). Current treatment primarily involves surgical resection of the bony bridge, with placement of an interpositional material (e.g. cranioplasty cements, cellulose-based matrices, autologous fat graft) to fill the gap. The optimal outcome would be prevention of bony bridge re-formation and regeneration of the native growth plate cartilage, restoring normal bone growth (123). However, the interpositional materials currently in use can either offer mechanical properties but lack the ability to integrate or biodegrade, or those that offer the potential for integration or degradation lack mechanical properties. Moreover, present materials lack the ability to modulate intrinsic pathways to prevent osteogenesis and promote cartilage repair. Because most treatment for bony bridge requires surgical resection and implantation of a material, it provides an avenue for improved, locally delivered therapeutics that may restore growth plate function. Novel therapeutic strategies can be tested for potential efficacy in small animals, such as rats or mice, while larger animals, such as rabbit, mini-pig or sheep are used to test performance in bony bar revision procedures more similar to those performed in patients (123).

The unique cellular environment and regulatory pathways of the growth plate make miRNA-based interventions a potential strategy to minimize adverse outcomes post-injury, by modulating signaling pathways involved in osteogenesis, inflammation, and chondrocyte stability. Further, during fracture repair, chondrocytes can transdifferentiate into osteoblasts; thus an understanding of the mechanisms underlying this process could also be beneficial for designing strategies to restrain bony bar formation (30, 124, 125) Several miRNAs, such as miR-140 and miR-145, are known to regulate chondrocyte differentiation by inhibiting osteogenic signaling, potentially offering a method to prevent abnormal bone formation within the growth plate after injury (19, 126, 127). By selectively targeting pro-osteogenic pathways, localized delivery of these miRNAs might help maintain a chondrogenic microenvironment, preserving the function of the growth plate and preventing bony bridge re-formation. Other miRNAs such as miR-374, miR-379 and miR-503 are crucial for enhancing chondrocyte proliferation; their delivery could foster a pro-regenerative environment by promoting chondrocyte growth and matrix stability without encouraging excessive bone formation (13). Clearly, a complete understanding of miRNA function in different zones of the growth plate could provide additional miRNAs to target for therapeutic intervention.

### 7.4 Challenges in miRNA delivery to cartilage tissue

Recent reviews have nicely summarized the various chemical modifications made to synthetic miRNA mimics and inhibitors that increase their stability, decrease immunogenicity, and improve cellular uptake. These reviews also discuss miRNA-based therapeutics currently in clinical trials (128, 129). Here, we limit our discussion to strategies most relevant to cartilage delivery.

The avascularity and dense extracellular matrix of cartilage pose significant barriers to drug delivery. Recent advances in nanoparticle and exosome-based delivery systems offer potential solutions, as nanoparticles and exosomes provide a vehicle for encapsulating miRNAs, enhancing their stability, and enabling their delivery to the dense cartilage matrix (130). These approaches are being optimized primarily in small animal models of osteoarthritis, where exosomes are administered via direct injection or via scaffold encapsulation for sustained release, which could improve targeting while minimizing off-target diffusion. For example, exosomes engineered with type II collagen-binding cartilage affinity peptides (CAPs) (e.g. DWRVIIPPRPSA) show enhanced retention in the joint, directing delivery of therapeutic miR-140 mimic to damaged cartilage, achieving better outcomes in preclinical osteoarthritis models (131). Additionally, novel cartilage-penetrating nanoparticles, such as CAP-PVAm-PLGA nanoparticles, were shown to penetrate ex vivo human cartilage to depths over 1000 µm, and could facilitate sustained release of therapeutic miRNAs such as miR-140 (132). Enhanced localization within the cartilage could address significant delivery challenges, potentially enabling precise treatment of growth plate injuries. Moreover, composite biomaterials incorporating nanoparticles that carry miRNA therapeutics in matrices tuned for specific mechanical properties and degradation rates may facilitate cartilage repair after bony bridge excision.

### 7.5 Potential risks and considerations in pediatric populations

Applying miRNA-based therapies in pediatric populations presents challenges due to the sensitivity of growth plates to hormonal and genetic signals. miRNA-based interventions could inadvertently suppress normal growth processes or have off-target effects, impacting other developmental pathways. Long-term effects of altering miRNA levels in children remain uncertain, posing potential risks to immune function, systemic development, or premature growth plate closure. Therefore, precision in miRNA dosage and delivery is crucial.

Indeed, although there are miRNA-based therapeutics in clinical trials, systemic delivery is problematic due to the exposure of non-target tissues to the therapeutic. Moreover, nucleic acids can activate toll-like receptors (TLRs) and trigger an interferon response, causing side effects. Titration of an appropriate miRNA dose is also critical because exogenous miRNAs can cause unexpected responses due to their competition with endogenous miRNAs for miRNA processing machinery and RISC components. High levels of exogenous miRNAs can also engage lower affinity targets, causing additional unanticipated effects (133). Pediatric applications necessitate further research to ensure safe, effective delivery that aligns with the unique physiological demands of the growing skeleton.

While miRNA-based therapeutics, especially those targeting miR-140, hold substantial potential for cartilage repair, significant challenges remain in adapting these treatments for clinical use in growth plate disorders. Advancements in delivery platforms such as exosome engineering and nanoparticle design, combined with a thorough understanding of the molecular mechanisms by which miRNAs regulate growth plate function will lay the groundwork for innovative treatments for injury to the growth plate and other cartilage compartments (86).
